# Treatment Effectiveness and Tolerability of Long-term Adjuvant First- and Second-Generation Epidermal Growth Factor Receptor Tyrosine Kinase Inhibitor at Different Doses in Patients With Stage IIA–IIIB Epidermal Growth Factor Receptor-Mutated Lung Adenocarcinoma: A Retrospective Study

**DOI:** 10.3389/fsurg.2022.816018

**Published:** 2022-03-11

**Authors:** Jing-Ren Ye, Pei-Hsing Chen, Jen-Hao Chuang, Mong-Wei Lin, Tung-Ming Tsai, Hsao-Hsun Hsu, Jin-Shing Chen

**Affiliations:** ^1^Division of Thoracic Surgery, Department of Surgery, National Taiwan University Hospital, Taipei, Taiwan; ^2^Division of Thoracic Surgery, Department of Surgery, National Taiwan University Hospital Yun-Lin Branch, Yun-Lin, Taiwan; ^3^Department of Surgical Oncology, National Taiwan University Cancer Center and National Taiwan University College of Medicine, Taipei, Taiwan

**Keywords:** adjuvant therapy, epidermal growth factor receptor-tyrosine kinase inhibitor, non-small-cell lung carcinoma, thoracic surgery, advanced lung cancer

## Abstract

**Introduction:**

For patients with epidermal growth factor receptor (EGFR)-mutated lung cancer who undergo surgery, adjuvant tyrosine kinase inhibitor (TKI) therapy other than osimertinib is an alternative option. We aimed to discuss the long-term safety and efficacy of TKI treatment in real-world data.

**Methods:**

From January 2011 to May 2020, patients with stage II–III EGFR-mutated adenocarcinoma who underwent cancer resection surgery at a single center were enrolled. The primary endpoint was disease relapse, and the secondary endpoint was overall survival. In total, 30 patients were included in the study. In our study, all patients underwent complete resection using video-assisted thoracoscopic surgery. The patients were divided into a dose interruption (prolonged interval use) group and non-dose adjustment group.

**Results:**

The patients' pathological stages were II–III. The initial EGFR TKIs were mostly gefitinib (*n* = 25, 83%), and others were erlotinib (*n* = 3, 10%) and afatinib (*n* = 2, 6%). The mean disease-free survival (DFS) was 53.3 months. The 2- and 5-year DFS rate was 90.0 and 73.3%, respectively. The median TKI treatment duration in this study was 44.5 months (range, 6–133 months), which was the longest in the literature review. Of these patients, nine had dose interruption. We compared the two groups and found no treatment differences between them. There were no significant side effect potentials between both groups.

**Conclusion:**

To our knowledge, this study provides the longest experience of TKI in patients with resected EGFR mutations and also provided a dose reduction strategy (prolonged medication interval) for patients who had intolerable side effects.

## Introduction

The epidermal growth factor receptor (EGFR) tyrosine kinase inhibitor (TKI) erlotinib has been proven to be effective in patients with advanced stage lung cancer (1). The adjuvant use of TKIs is also important in patients who cannot tolerate the toxicity of chemotherapy and their use might improve the outcomes of a subset of patients. Some studies have suggested that TKIs are more effective in selected East Asian patients than carboplatin–paclitaxel as an initial treatment (2).

However, the efficacy and safety of adjuvant EGFR TKI therapy in patients with resected lung cancer is still debated, although prospective randomized controlled trials (3–5) have shown improved disease-free survival results for adjuvant EGFR TKI use in patients with resected lung cancer. The single-arm trial SELECT (6) also indicated positive results for adjuvant EGFR TKI use compared to historical data.

Previous articles on treatment duration all had a short duration of treatment (<20 months except in two articles), which was not long enough for the effects of the long-term use of first-line TKI to be observed. The shortest median treatment duration of BR19 was 4.8 months. The outcome of BR19 might be too short to evaluate the adjuvant TKI usage, and the number of patients with EGFR mutations was low at only 4% (15 cases of tumor) in the cohort. The treatment duration of gefitinib for ADJUVANT was 21.9 months. The longest duration of TKI treatment was the ADAURA trial, which used the third-generation TKI, osmertinib, for 36 weeks. The treatment duration of the RADIANT study was 11.9 months, but in the EGFR mutation subgroup, the median treatment duration was 21.2 months. To improve the outcome of TKI treatment, long-term use of TKIs has become an important issue.

In cases of long-term use of TKIs, side effects are usually encountered. Rashes, diarrhea, and elevated liver function tests were listed most frequently in studies. In the ADAURA trial, a third-generation TKI was used, and the side effects were still high. A total of 97% of the patients had side effects. From the above study, we know that above 50% of patients would have side effects if postoperative adjuvant TKI is administered.

Therefore, the strategy of managing the medication side effects is the next important issue. The strategy of managing the side effects of medication include dose reduction or medication shift. However, a retrospective analysis with a reduced dose showed a progression-free survival (PFS) of 8.8 months compared to 11.2 in those who received the full dose (7). Another *post-hoc* analysis for afatinib showed that patients whose doses were reduced to 30 mg in the first 6 months had acceptable PFS of 11.3 months compared to 11.0 months in those who did not receive dose reduction (8).

However, results on the survival and adverse outcomes even after long-term use of adjuvant EGFR TKI in patients with stage IIA–IIIB lung cancer are still inadequate. Herein, we retrospectively reviewed patients with resected non-small-cell lung adenocarcinoma who were treated with adjuvant EGFR TKI for nearly a decade at a single center with the aim to develop a long-term treatment strategy.

## Patients and Methods

### Study Design and Patient Population

**Figure 1:**
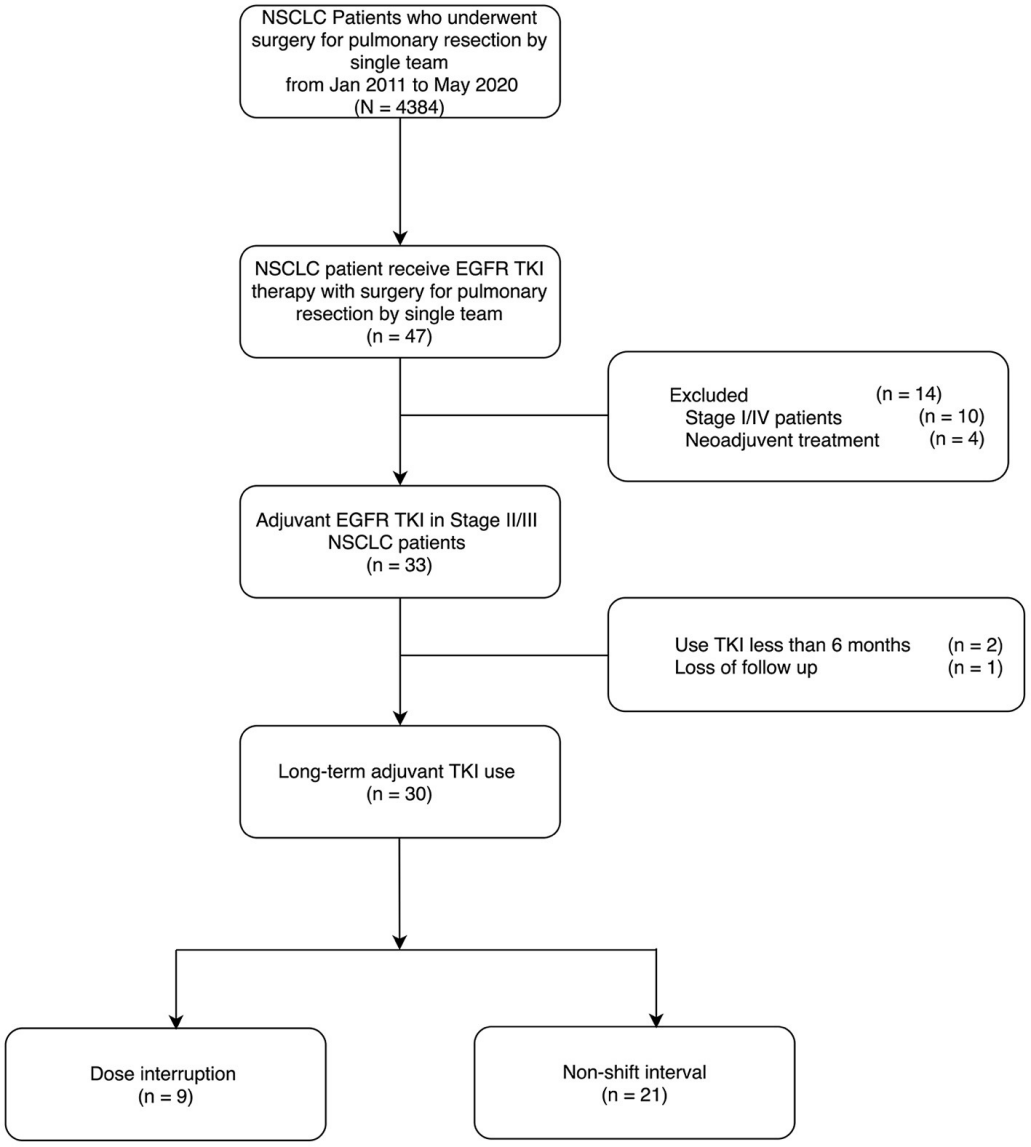
Eligible criteria.

We retrospectively reviewed patients with resected non-small-cell lung adenocarcinoma who were treated with adjuvant EGFR TKI between January 2011 and May 2020. The eligible patients were those aged ≥ 18 years who had completely resected NSCLC with stage IIA to IIIB NSCLC with EGFR mutation, followed by an adjuvant TKI therapy of 6 months or more. The TKIs prescribed in our study included gefitinib, erlotinib, and afatinib. To evaluate a long-term follow-up of TKI use, patients who received an adjuvant TKI treatment duration of 6 months or more were included. Patients who received chemotherapy or any form of neoadjuvant therapy were excluded from our study. Patients who had undergone previous lung cancer surgery were also excluded (Figure 1). The cancer stage of the data was documented using the American Joint Committee on Cancer 8th edition staging system.

Our clinical patient-care protocol in this study included adjuvant TKI treatment 1 month after the operation, and the patients would receive adjuvant TKI treatment for as long as possible without a limited treatment course. All patients underwent complete surgical resection. The average daily dose was calculated by dividing the sum of all doses of prescribed TKI tablets by the total number of days of treatment. All patients with intolerable side effects were recorded in the patients' charts and graded using the Common Terminology Criteria for Adverse Events (version 3). When an adverse event develops, the patient was treated conservatively with medication for symptom relief and the temporary suspension of TKI medication if needed. Patients who can be treated conservatively were classified into “non-shift interval” group. However, a reduction of dosage or frequency or TKI medication shift was implemented if the patient experienced prolonged side effects without improvement after conservative treatment or intolerance by the patient, such as hepatotoxicity, skin rash, or nausea. In such cases, the patients were classified in the “dose interruption” group. Temporary holding of TKI was not considered as a dose interruption.

During the follow-up period of adjuvant TKI treatment, the patients needed monthly check of laboratory data, including liver function and carcinoembryonic antigen. Besides, the patients also received routine image studies, including computed tomography (CT) of the brain, chest, and abdomen every 3 months. Magnetic resonance imaging of the brain, bone scan, or positron emission tomography scan was optional if needed. Recurrence was confirmed by either imaging or histopathological diagnosis of biopsy.

### Statistical Analysis

The primary endpoint was disease-free survival. The secondary endpoint was overall survival. Kaplan–Meier curves were used to describe the time-to-event. Log-rank tests were used to evaluate the differences in disease-free survival (DFS) between the patient groups. Survival was counted from the day after surgery. Continuous variables were presented as mean and median (range), and categorical variables were presented as numbers and percentages for analysis. The statistical differences between the responses among the patient groups at different daily doses were determined using the χ^2^ test or Fisher's exact test for categorical variables. All tests for significance were two-sided. Statistical significance was set at *p* < 0.05. All analyses were performed using the SPSS software (version 26 for Mac, SPSS Inc., Chicago, IL, USA). All data were followed up to March, 2021.

## Results

### Patient Characteristics

A total of 4,384 patients with primary lung cancer resection performed by a single team from January 2011 to May 2020 were consecutively recorded, and fortyseven patients of them received EGFR TKI therapy. Seventeen patients were excluded due to the reasons including stage I or IV (*n* = 10), receiving neoadjuvant therapy (*n* = 4), TKI therapy <6 months (*n* = 2), and loss of follow up (*n* = 1). Finally, a total of 30 patients were included as long-term adjuvant TKI use. All of these patients underwent complete lung cancer resection using VATS [IIA = 1 (3%) IIB = 3 (10%) IIIA = 19 (63%) IIIB = 7 (23%)]. The initial EGFR TKIs in our study were mostly gefitinib in 25 (83.3%) patients; erlotinib was used in three (10.0%) patients and afatinib in two (6.7%) patients. The median duration of TKI therapy was 44.5 months (Mean = 52.7 ± 32.0 months), which were ranged from 6 to 133 months (Table 1). Seventy percent of patients (N = 21) did not require a permanent dosage reduction or medication shift (non-shift interval group). However, 30% of patients (*N* = 9) required a medication shift because of intolerance of the side effects (dose interruption group). All of the medication shifts in our study were from gefitinib to erlotinib.

**Table 1:** Patient characteristics.

**Characteristic**	**Number (%)**			***p*-value**
	**Adjuvant TKI (*n* = 30)**	**Dose interruption (*n* = 9)**	**Non-shift interval (*n* = 21)**	
Sex	–	–	–	–
Male	9 (30%)	5 (17%)	4 (13%)	0.082
Female	21 (70%)	4 (13%)	17 (56%)	0.082
Age	–	–	–	–
Mean ± SD	65.3 ± 11.7	68.8 ± 11.3	63.8 ± 11.8	–
Median (Min to Max)	65.5 (40 to 83)	68 (48 to 81)	63 (40 to 83)	–
Smoking	–	–	–	–
Never	28 (93%)	7 (23%)	21 (70%)	0.083
Former	1 (3%)	1 (3%)	0 (0%)	0.300
Current	1 (3%)	1 (3%)	0 (0%)	0.300
Pathologic stage (AJCC 8th)	–	–	–	–
Stage II	4 (13%)	1 (3%)	3 (10%)	>0.999
Stage IIA	1 (3%)	1 (3%)	0 (0%)	0.300
Stage IIB	3 (10%)	0 (%)	3 (10%)	0.535
Stage III	26 (86%)	8 (27%)	18 (60%)	>0.999
Stage IIIA	19 (63%)	5 (16%)	14 (46%)	0.687
Stage IIIB	7 (23%)	3 (10%)	4 (13%)	0.640
EGFR TKI Medication	–	–	–	–
Gefitinib	25 (83%)	4 (13%)	12 (40%)	0.694
Erlotinib	3 (10%)	1 (3%)	2 (7%)	>0.999
Afatinib	2 (%)	0 (%)	2 (7%)	>0.999
Medication shift	9 (30%)	4 (13%)	5 (17%)	0.389
Mutation Type	–	–	–	–
Exon 21 L858R	14 (46%)	6 (20%)	8 (27%)	0.109
Exon 19	11 (37%)	2 (6%)	9 (30%)	0.262
Exon 18	1 (3%)	0 (%)	1 (3%)	>0.999
Rare mutation	4 (12%)	1 (%)	3 (10%)	>0.999
Disease Progression Site	–	–	–	–
Brain	5 (17%)	4 (10%)	1 (13%)	0.19
Lymph node progression	1 (3%)	0 (0%)	1 (3%)	>0.999
Pleural seeding	1 (3%)	0 (0%)	1 (3%)	>0.999
Lung to lung metastasis	1 (3%)	1 (3%)	0 (0%)	>0.999
Duration of treatment (months)	–	–	–	–
Mean ± SD	52.7 ± 32.0	–	–	–
Median (Min to Max)	44.5 (6~133)	–	–	–

**Figure 2:**
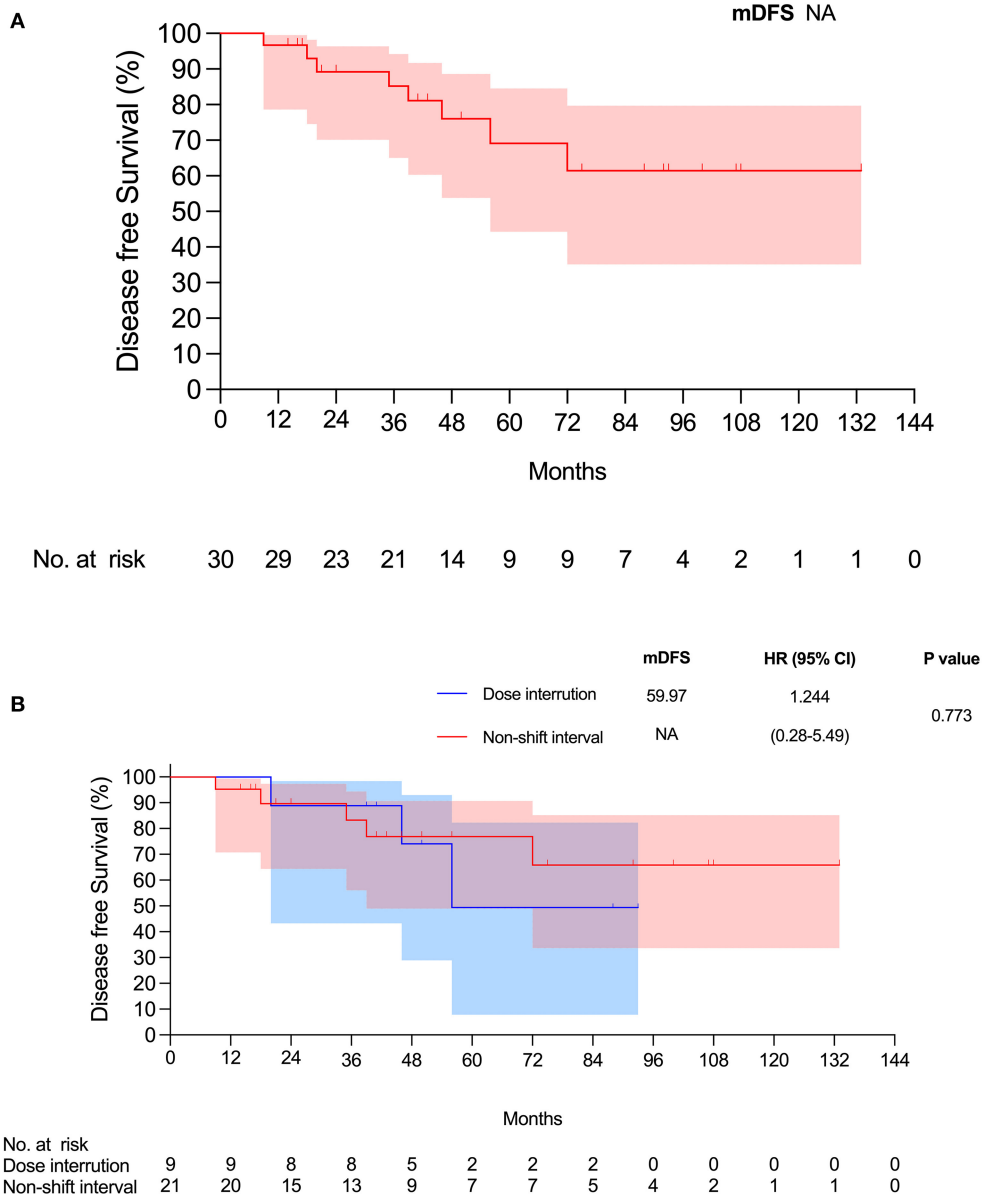
**(A)** Disease-free survival for all patients. **(B)** Disease-free survival for two groups.

**Figure 3:**
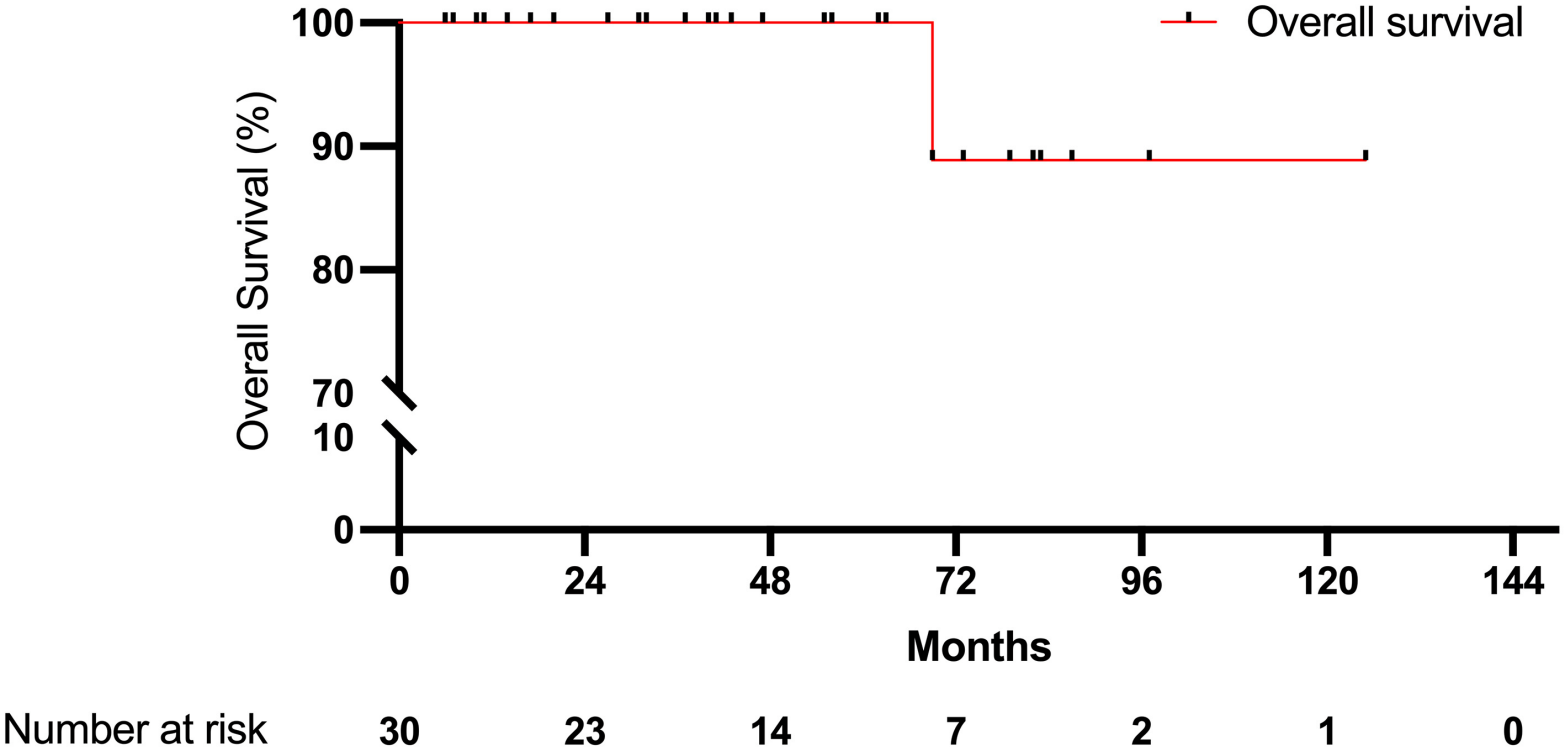
Overall survival.

The median follow-up time of all 30 patients was 45 months. The follow-up time was the same as the duration of TKI therapy in the disease-free patients because of our study design. The mean disease-free survival (DFS) was 53.3 months. Five patients (17%) had disease progression. The 2-year DFS rate was 90%, which included only 21 patients at the end of the 2-year follow-up period. The 5-year DFS rate was 73.3%, which included nine patients at the end of the 5-year follow-up. For overall survival, only one patient (3%) died during follow-up at 69 months; thus, the 2-year overall survival rate was 100% and included 23 patients at the end of the 2-year follow-up. The 5-year overall survival was still 100% and included 11 patients at the end of the 5-year follow-up. The results are demonstrated in Figures 2, 3.

### Treatment Effectiveness

We compared the groups of patients who took medication with and without a dose interruption (dose interruption group and non-shift interval group). We analyzed the data using Kaplan–Meier survival curves, which showed no statistically significant difference in disease-free survival and overall survival between the two groups (Figures 2, 3). The median disease-free survival with dose interruption was 60.0 months, and the disease-free survival for the non-shift group was immature. The hazard ratio for the dose interruption group and the non-shift group was 1.244 (0.28–5.49), with a *p*-value of 0.773. Five patients had recurrence with brain metastasis, one patient had pleural seedings, one patient had lung-to-lung metastasis, and one patient had lymph node metastasis (Table 1). Four of the five patients with brain metastasis had dose interruption, which is not statistically significant when compared with patients without dose interruption (*p* = 0.19). Medication interruption, sex, age, smoking history, stage, and mutation type were not risk factors of disease-free survival in the logistic regression models (Table 2).

**Table 2:** Risk factors of disease-free survival.

**Variable**	**Univariate analysis**	***p*-value**
	**HR (95% CI)**	
Medication interruption (without vs. with)	1.235 (0.292~5.221)	0.774
Sex (male vs. female)	1.001 (0.199~5.026)	0.999
Age (≥65 vs. <65 years)	3.261 (0.651~16.346)	0.151
Smoking history (ever smokers vs. never smokers)	1.242 (0.150~10.252)	0.841
Stage	1.256 (0.153~10.284)	0.832
Mutation type Exon 19 vs. L858R	2.41 (0.437~13.286)	0.313
Mutation type Exon 19 vs. others	3.074 (0.430~21.988)	0.263

### Adverse Event and Adverse Event Management

In our study, most of the side effects of medications were skin rashes (53.3%) and elevated liver function test results (46 and 66%, respectively). Other side effects include paronychia, anemia, nausea, and diarrhea (Table 3). There were no grade III or higher side effects, and no patient had irreversible outcomes such as fibrosis in our study.

**Table 3:** Maximal adverse event for the dose interruption and non-shift interval groups.

**Adverse event**	**Arm (*****n*** **=** **30)**				***p*-value**
	**Dose interruption** **(*****n*** **=** **9)**		**Non-shift interval** **(*****n*** **=** **21)**		
	**Any adverse event**	**CTC grade 2, 3, 4, or 5**	**Any adverse event**	**CTC grade 2, 3, 4, or 5**	
Rash	6 (20%)	3 (10%)	10 (33%)	3 (10%)	0.44
Elevated ALT	8 (26%)	2 (6%)	12 (40%)	6 (20%)	0.20
Elevated AST	9 (30%)	2 (6%)	11 (36%)	0 (0%)	0.01
Dry skin	3 (10%)	0 (0%)	1 (3%)	0 (0%)	0.11
Paronychia	0 (0%)	0 (0%)	1 (3%)	1 (3%)	>0.99
Anemia	0 (0%)	0 (0%)	1 (3%)	0 (0%)	>0.99
Nausea	0 (0%)	0 (0%)	1 (3%)	0 (0%)	>0.99
Diarrhea	1 (3%)	0 (0%)	1 (3%)	0 (0%)	0.52
Oral ulcer	0 (0%)	0 (0%)	1 (3%)	0	>0.99

The most common reason for dose interruption was safety/tolerability (88.9%). Another reason was the investigator's decision (11.1%). A total of nine patients had interruption of medication, and another nine had medication shift. Four patients in the dose interruption group first experienced a medication shift. Another five patients received a medication shift without dose interruption. Two (6.7%) patients were taking medication for several months and then resumed. The adverse event with dose interruption showed no significant differences between the groups without medication interruption. All patients who underwent dose intervention or medication shifts did so within 20 months. All adverse events were stable in the first 20 months (Table 4).

**Table 4:** Categories of dose interruption (interval change).

**Variable**	**All patients**
Interval change event	–
No change	21 (70.0%)
Average 50.0% daily dose	6 (20.0%)
Average 66.6% daily dose	1 (3.3%)
Average 50.0% daily dose tapper to average 33.3% daily dose	1 (3.3%)
Average 50.0% daily dose tapper to average 66.6% daily dose	1 (3.3%)

## Discussion

To the best of our knowledge, this article provides the longest real-world data of long-term adjuvant TKI therapy in patients with resected NSCLC harboring activating EGFR mutations. This article is a real-world retrospective article. In addition, we demonstrated that subgroup analysis for patients who received first- and second-generation EGFR TKIs (mainly initiated from gefitinib or erlotinib) medication interruption in the real-world data had similar DFS, OS, and adverse event rates compared to those without medication interval change as their long-term treatment dose in patients had adverse effect.

Adjuvant therapy has been researched and is intended to improve survival after microscopically margin-negative resection (R0) or complete tumor resection. The standard treatment was chemotherapy after the 2003 presentation of the International Adjuvant Lung Cancer Trial (9). Several adjuvant protocols have been used to improve outcomes after surgery. A study on the addition of bevacizumab to four cycles of adjuvant cisplatin-based chemotherapy in patients with resected early-stage NSCLC was named E1505. However, there was still no improvement in OS or PFS in the E1505 study (10). TKIs such as gefitinib, erlotinib, and afatinib were used as adjuvant therapy in cases of suspected micrometastatic disease postoperatively; these patients would benefit from TKI since it could cure the micrometastatic cancer even if the primary tumor had been resected (11). A meta-analysis (12) evaluated EGFR TKIs in patients who received adjuvant therapy. The study showed a statistically significant improvement in DFS for a 52% reduction in operable NSCLC harboring activating EGFR mutations. Moreover, there was a reduction in distant metastasis (not reaching statistical significance).

Three prospective randomized controlled trials (ADJUVANT, EVAN, and ADAURA) showed the effectiveness of adjuvant TKI treatment (3–6) in operable NSCLC patients harboring activating EGFR mutations. Compared with previous studies, two RCTs focused on patients with EGFR mutations. There is a doubt that the data from non-selected patients with EGFR mutations may be misleading. Therefore, misleading factors were excluded in these two RCTs. In the RADIANT study, the EGFR mutation subgroup was selected. In the ADJUVANT, EVAN, and RADIANT EGFR mutation subgroups, all three groups showed improved PFS with adjuvant TKI use.

There is a debate regarding the optimal treatment duration of adjuvant EGFR-TKI. Since we understand that TKI can not eradicate all EGFR positive cancer cells in stage IV lung cancer, a prolonged EGFR TKIs exposure may result in significant benefits. The median TKI treatment duration was 11.9 months in RADIANT and 23.9 months in EVAN. In a more recent double-blind, phase 3 trial (ADURA), the TKI treatment duration was designed to be 36 months (5), and the results showed improved PFS without overall survival benefit. This trial was also the longest; however, the effectiveness and tolerability after long-term use are unclear and without real-world data. We had data for up to 133 months with a median 44.5 months of TKI medication use. There was no elevation of adverse event rate after long-term use of adjuvant TKIs (Table 1 and Table 3). Nevertheless, the optimal adjuvant TKI duration should balance the side effects with the benefits of treatment.

However, adverse drug reactions (ADRs) have been reported in 4–11% of patients taking first- and second-generation EGFR TKIs (13). A recent large meta-analysis of 2,535 patients revealed that approximately 40% of patients taking first- and second-generation EGFR-TKIs developed grade 3–4 ADRs. Our analysis showed that the risk of grade 3–4 ADRs was higher for afatinib (42.1%) and erlotinib (54.1%) than gefitinib (29.1%). In addition, nearly 20% of the patients initially taking afatinib 40 mg had a reduced dose due to ADRs. However, the lower starting dose with afatinib 30 mg daily showed no reduction in dose or discontinuation of medication (14). Real-world data of first-line afatinib treatment showed that dose reduction occurred in 47.5–76.3% of cases (15, 16).

In addition, the LUX-Lung 6 and LUX-Lung 3 trials showed dose reductions of 28.0% (67/239) and 53.3% (122/229), respectively, when patients received a daily dose of 40 mg afatinib. The timing of the dose reduction event developed mostly within the first 6 months. Reducing the dose of afatinib decreased the incidence of ADRs but retained a similar median PFS based on the subgroup analyses performed in the LUX-Lung 3 and LUX-Lung 6 trials (8, 17, 18). EGFR TKIs were considered to have a predictable adverse event (AE) profile, but with differences among different EGFR TKIs. Afatinib has a clear dose adjustment protocol for the management of treatment-related AEs but maintains a similar efficacy (8, 19). A phase II study assessing the effect of malnutrition and BSA on afatinib-related AEs survival outcome found that PFS was not significantly different in patients with or without dose reduction [median 9.2 vs. 14.6 months (*P* = 0.337)] (20).

Review of previous article on adjuvant TKI therapy, dose reductions or interruptions were not well-investigated in studies yet. Only a few small studies have suggested dose reductions in these agents. A study of octogenarians (*n* = 21) receiving erlotinib or gefitinib showed that 70% of the patients required dose reduction (21). Gefitinib and erlotinib therapy may be beneficial in patients aged ≥80 years, and EGFR TKI dose modification may be necessary according to the overall medical condition of elderly patients (21). A retrospective study assessed the effects of full-dose erlotinib administration (*n* = 172) vs. reduced-dose erlotinib administration (*n* = 34); having a lower starting dose did not significantly affect efficacy (median, 8.8; 11.2 months; HR, 0.75; *P* = 0.14) (7).

Several studies have shown that dose reduction or gefitinib/erlotinib dose interruption (e.g., dosing every other day) did not adversely affect efficacy (22–29). However, real-world data for inoperable/recurrent EGFR mutation-positive NSCLC have been reported (30, 31). However, the long-term effect in practice, especially in the early-stage adjuvant treatment group, is not well-established. In our study, most patients were taking gefitinib or erlotinib (93.3%). When ADRs developed, the patients might not tolerate the adverse effect, so the medication strategy change is inevitable. In our study, medication change (*n* = 5), medication interruption (*n* = 5), or both (*n* = 4) were applied to the patients. The long-term survival results showed no significant difference in DFS or OS.

The SELECT study indicated that adjuvant TKI usage does not lead to a resistant disease. Sixty-five percent of recurrence cases were retreated and the treatment duration (13.1 months) approximated the PFS of erlotinib in a *de novo* metastatic EGFR-mutant population, which implied that the long-term adjuvant TKI could be safe and that the tumor would still be sensitive to EGFR TKIs even after relapse following adjuvant erlotinib use.

Our results showed that treatment with TKI for up to 29 months was tolerable. In RADIANT, the patient was allowed to receive two dose reductions because early-stage patients were often unwilling to tolerate side effects. For the reduced dose, there were no prospective randomized trials in patients with NSCLC. Compared with the previous study, we did not reduce the daily dose but used medication interruption (shifted the frequency). In our study, although the study population was relatively small, the follow-up time was long enough to allow us to observe the outcome in the subgroup. Treatment outcome was acceptable in the medication interruption group.

We acknowledge several limitations of this study. First, as a retrospective, single-institution analysis, time-trend bias and patient selection bias were inevitable. Secondly, the study population was exclusively eastern Asian, with a uniquely high proportion of female non-smokers and EGFR mutations. Clinical application to other NSCLC populations should be made with caution. Thirdly, the small number of cases is another limitation. Finally, adjuvant TKI in stage IIA-IIIB lung cancer without chemotherapy is not a standard option of care, and our results should be validated in a more extensive multi-institutional randomized study in the future.

In conclusion, this study provided the longest experience, to our knowledge, of TKI use in patients with resected EGFR mutations. Long-term use of TKIs could be considered a safe option with fewer side effects in adjuvant treatment settings.

## Data Availability Statement

The original contributions presented in the study are included in the article/supplementary material, further inquiries can be directed to the corresponding authors.

## Ethics Statement

The studies involving human participants were reviewed and approved by National Taiwan University Hospital. Written informed consent for participation was not required for this study in accordance with the national legislation and the institutional requirements.

## Author Contributions

J-RY and P-HC: manuscript writing, statistics, and data collection. J-HC: data collection. M-WL and H-HH: supervise and organize. T-MT: corresponding, data collection, and organize. J-SC: concept, organize, and supervise. All authors contributed to the article and approved the submitted version.

## Funding

This work was supported in part by research grants from National Taiwan University Hospital and the Taiwan Lung Foundation, Taipei, Taiwan. The funder had no role in the study design, data collection, analysis, decision to publish, or preparation of the manuscript.

## Conflict of Interest

The authors declare that the research was conducted in the absence of any commercial or financial relationships that could be construed as a potential conflict of interest.

## Publisher's Note

All claims expressed in this article are solely those of the authors and do not necessarily represent those of their affiliated organizations, or those of the publisher, the editors and the reviewers. Any product that may be evaluated in this article, or claim that may be made by its manufacturer, is not guaranteed or endorsed by the publisher.

## References

[1] Osarogiagbon RU Cappuzzo F Ciuleanu T Leon L Klughammer B. Erlotinib therapy after initial platinum doublet therapy in patients with EGFR wild type non-small cell lung cancer: results of a combined patient-level analysis of the NCIC CTG BR. 21 and SATURN trials. Transl Lung Cancer Res. (2015) 4:465–74. 10.3978/j.issn.2218-6751.2015.07.17 26380188 PMC4549481

[2] Mok TS Wu YL Thongprasert S Yang CH Chu DT Saijo N. Gefitinib or carboplatin-paclitaxel in pulmonary adenocarcinoma. N Engl J Med. (2009) 361:947–57. 10.1056/NEJMoa0810699 19692680

[3] Kelly K Altorki NK Eberhardt WE O'Brien ME Spigel DR Crinò L. Adjuvant erlotinib versus placebo in patients with stage IB-IIIA non-small-cell lung cancer (RADIANT): a randomized, double-blind, phase III trial. J Clin Oncol. (2015) 33:4007–14. 10.1200/JCO.2015.61.8918 26324372

[4] Zhong WZ Wang Q Mao WM Xu ST Wu L Shen Y. Gefitinib versus vinorelbine plus cisplatin as adjuvant treatment for stage II-IIIa (N1-N2) EGFR-mutant NSCLC (ADJUVANT/CTONG1104): a randomised, open-label, phase 3 study. Lancet Oncol. (2018) 19:139–48. 10.1016/S1470-2045(17)30729-5 29174310

[5] Wu YL Herbst RS Mann H Rukazenkov Y Marotti M Tsuboi M. ADAURA: phase III, double-blind, randomized study of osimertinib versus placebo in EGFR mutation-positive early-stage NSCLC after complete surgical resection. Clin Lung Cancer. (2018) 19:E533–E6. 10.1016/j.cllc.2018.04.004 29789220

[6] Pennell NA Neal JW Chaft JE Azzoli CG Jänne PA Govindan R. SELECT: a phase II trial of adjuvant erlotinib in patients with resected epidermal growth factor receptor-mutant non-small-cell lung cancer. J Clin Oncol. (2019) 37:612. 10.1200/JCO.18.00131 30444685 PMC6524649

[7] Lampson BL Santos A Janne PA Oxnard GR. Reduced-dose versus full-dose erlotinib for advanced EGFR-mutant non-small cell lung carcinoma (NSCLC): a retrospective analysis. J Clin Oncol. (2015) 33:8074. 10.1200/jco.2015.33.15_suppl.8074

[8] Yang JC Sequist LV Zhou C Schuler M Geater SL Mok T. Effect of dose adjustment on the safety and efficacy of afatinib for EGFR mutation-positive lung adenocarcinoma: Post hoc analyses of the randomized LUX-Lung 3 and 6 trials. Ann Oncol. (2016) 27:2103–10. 10.1093/annonc/mdw322 27601237

[9] Arriagada R Bergman B Dunant A Le Chevalier T Pignon JP Vansteenkiste J. Cisplatin-based adjuvant chemotherapy in patients with completely resected non-small-cell lung cancer. N Engl J Med. (2004) 350:351–60. 10.1056/NEJMoa031644 14736927

[10] Wakelee HA Dahlberg SE Keller SM Tester WJ Gandara DR Graziano SL. Adjuvant chemotherapy with or without bevacizumab in patients with resected non-small-cell lung cancer (E1505): an open-label, multicentre, randomised, phase 3 trial. Lancet Oncol. (2017) 18:1610–23. 10.1016/S1470-2045(17)30691-5 29129443 PMC5789803

[11] Janjigian YY Park BJ Zakowski MF Ladanyi M Pao W D'Angelo SP. Impact on disease-free survival of adjuvant erlotinib or gefitinib in patients with resected lung adenocarcinomas that harbor EGFR mutations. J Thorac Oncol. (2011) 6:569–75. 10.1097/JTO.0b013e318202bffe 21150674 PMC3778680

[12] Huang Q Li J Sun Y Wang R Cheng X Chen H. Efficacy of EGFR tyrosine kinase inhibitors in the adjuvant treatment for operable non-small cell lung cancer by a meta-analysis. Chest. (2016) 149:1384–92. 10.1016/j.chest.2015.12.017 26836897

[13] Park K Tan EH O'Byrne K Zhang L Boyer M Mok T. Afatinib versus gefitinib as first-line treatment of patients with EGFR mutation-positive non-small-cell lung cancer (LUX-Lung 7): a phase 2B, open-label, randomised controlled trial. Lancet Oncol. (2016) 17:577–89. 10.1016/S1470-2045(16)30033-X 27083334

[14] Ding PN Lord SJ Gebski V Links M Bray V Gralla RJ. Risk of treatment-related toxicities from EGFR tyrosine kinase inhibitors: a meta-analysis of clinical trials of gefitinib, erlotinib, and afatinib in advanced EGFR-mutated non-small cell lung cancer. J Thorac Oncol. (2017) 12:633–43. 10.1016/j.jtho.2016.11.2236 28007626

[15] Imai H Kaira K Suzuki K Anzai M Tsuda T Ishizuka T. A phase II study of afatinib treatment for elderly patients with previously untreated advanced non-small-cell lung cancer harboring EGFR mutations. Lung Cancer. (2018) 126:41–7. 10.1016/j.lungcan.2018.10.014 30527191

[16] Tanaka H Taima K Itoga M Ishioka Y Baba K Shiratori T. Real-world study of afatinib in first-line or re-challenge settings for patients with EGFR mutant non-small cell lung cancer. Med Oncol. (2019) 36:57. 10.1007/s12032-019-1278-9 31089973

[17] Wu YL Zhou C Hu CP Feng J Lu S Huang Y. Afatinib versus cisplatin plus gemcitabine for first-line treatment of Asian patients with advanced non-small-cell lung cancer harbouring EGFR mutations (LUX-Lung 6): an open-label, randomised phase 3 trial. Lancet Oncol. (2014) 15:213–22. 10.1016/S1470-2045(13)70604-1 24439929

[18] Yang JC Wu YL Schuler M Sebastian M Popat S Yamamoto N. Afatinib versus cisplatin-based chemotherapy for EGFR mutation-positive lung adenocarcinoma (LUX-Lung 3 and LUX-Lung 6): analysis of overall survival data from two randomised, phase 3 trials. Lancet Oncol. (2015) 16:141–51. 10.1016/S1470-2045(14)71173-8 25589191

[19] Girard N. Optimizing outcomes in EGFR mutation-positive NSCLC: which tyrosine kinase inhibitor and when? Future Oncol. (2018) 14:1117–32. 10.2217/fon-2017-0636 29336166

[20] Arrieta O De la Torre-Vallejo M López-Macías D Orta D Turcott J Macedo-Pérez E-O. Nutritional status, body surface, and low lean body mass/body mass index are related to dose reduction and severe gastrointestinal toxicity induced by afatinib in patients with non-small cell lung cancer. Oncologist. (2015) 20:967–74. 10.1634/theoncologist.2015-0058 26173839 PMC4524769

[21] Nakao M Muramatsu H Sone K Aoki S Akiko H Kagawa Y. Epidermal growth factor receptor-tyrosine kinase inhibitors for non-small-cell lung cancer patients aged 80 years or older: a retrospective analysis. Mol Clin Oncol. (2015) 3:403–7. 10.3892/mco.2014.453 25798276 PMC4360513

[22] Chen YC Tsai MJ Lee MH Kuo CY Shen MC Tsai YM. Lower starting dose of afatinib for the treatment of metastatic lung adenocarcinoma harboring exon 21 and exon 19 mutations. BMC Cancer. (2021) 21:495. 10.1186/s12885-021-08235-3 33941115 PMC8091516

[23] Wei YF Lim CK Tsai MS Huang MS Chen KY. Intracranial responses to afatinib at different doses in patients with EGFR-mutated non-small-cell lung carcinoma and brain metastases. Clin Lung Cancer. (2019) 20:e274–e83. 10.1016/j.cllc.2019.02.009 30930121

[24] Yang CJ Tsai MJ Hung JY Lee MH Tsai YM Tsai YC. The clinical efficacy of afatinib 30 mg daily as starting dose may not be inferior to afatinib 40 mg daily in patients with stage IV lung adenocarcinoma harboring exon 19 or exon 21 mutations. BMC Pharmacol Toxicol. (2017) 18:82. 10.1186/s40360-017-0190-1 29237484 PMC5729426

[25] Halmos B Tan E-H Soo RA Cadranel J Lee MK Foucher P. Impact of afatinib dose modification on safety and effectiveness in patients with EGFR mutation-positive advanced NSCLC: results from a global real-world study (RealGiDo). Lung Cancer. (2019) 127:103–11. 10.1016/j.lungcan.2018.10.028 30642537

[26] Lim CK Wei YF Tsai MS Chen KY Shih JY Yu CJ. Treatment effectiveness and tolerability of afatinib at different doses in patients with EGFR-mutated lung adenocarcinoma: how low can we go? Eur J Cancer. (2018) 103:32–40. 10.1016/j.ejca.2018.07.128 30199768

[27] Sim SH Keam B Kim DW Kim TM Lee SH Chung DH. The gefitinib dose reduction on survival outcomes in epidermal growth factor receptor mutant non-small cell lung cancer. J Cancer Res Clin Oncol. (2014) 140:2135–42. 10.1007/s00432-014-1768-2 25005787 PMC11823989

[28] Sato S Kurishima K Miyazaki K Kodama T Ishikawa H Kagohashi K. Efficacy of tyrosine kinase inhibitors in non-small-cell lung cancer patients undergoing dose reduction and those with a low body surface area. Mol Clin Oncol. (2014) 2:604–8. 10.3892/mco.2014.281 24940504 PMC4051560

[29] Satoh H Inoue A Kobayashi K Maemondo M Oizumi S Isobe H. Low-dose gefitinib treatment for patients with advanced non-small cell lung cancer harboring sensitive epidermal growth factor receptor mutations. J Thorac Oncol. (2011) 6:1413–7. 10.1097/JTO.0b013e31821d43a8 21681118

[30] Tamura K Nukiwa T Gemma A Yamamoto N Mizushima M Ochai K. Real-world treatment of over 1600 Japanese patients with EGFR mutation-positive non-small cell lung cancer with daily afatinib. Int J Clin Oncol. (2019) 24:917–26. 10.1007/s10147-019-01439-5 30953238 PMC6597604

[31] Inoue A Yoshida K Morita S Imamura F Seto T Okamoto I. Characteristics and overall survival of EGFR mutation-positive non-small cell lung cancer treated with EGFR tyrosine kinase inhibitors: a retrospective analysis for 1660 Japanese patients. Jpn J Clin Oncol. (2016) 46:462–67. 10.1093/jjco/hyw014 26977054 PMC4874470

